# A novel prognostic model for Japanese patients with newly diagnosed bone‐metastatic hormone‐naïve prostate cancer

**DOI:** 10.1002/bco2.46

**Published:** 2020-09-18

**Authors:** Yasuhide Miyoshi, Masato Yasui, Shuko Yoneyama, Takashi Kawahara, Yoshihiro Nakagami, Yoshimasa Ohno, Junpei Iizuka, Kazunari Tanabe, Yasunobu Hashimoto, Hideyasu Tsumura, Ken‐ichi Tabata, Masatsugu Iwamura, Akihiro Yano, Satoru Kawakami, Hiroji Uemura

**Affiliations:** ^1^ Department of Urology and Renal Transplantation Yokohama City University Medical Center Yokohama Japan; ^2^ Department of Urology Tokyo Medical University Tokyo Japan; ^3^ Department of Urology Tokyo Women's Medical University Tokyo Japan; ^4^ Department of Urology Saiseikai Kawaguchi General Hospital Kawaguchi Japan; ^5^ Department of Urology Kitasato University School of Medicine Sagamihara Japan; ^6^ Department of Urology Saitama Medical Center Saitama Medical University Kawagoe Japan; ^7^ Department of Urology Showa University Tokyo Japan

## Abstract

**Objectives:**

To evaluate the prognosis of newly diagnosed patients with metastatic hormone‐naïve prostate cancer (mHNPC) and develop a novel prognostic model based on ChemoHormonal Therapy Versus Androgen Ablation Randomized Trial for Extensive Disease in Prostate Cancer (CHAARTED) risk classifications.

**Patients and methods:**

We retrospectively analyzed the data of 578 newly diagnosed mHNPC patients initially treated with androgen deprivation therapy. We evaluated three clinical factors, namely, CHAARTED risk classifications (high‐volume disease [HVD] vs low‐volume disease [LVD]), Gleason scores (GS, 9‐10 vs ≤8), and hemoglobin (Hb, ≤13.0 g/dL vs >13.0 g/dL), for their prognostic potential in predicting time to castration‐resistant prostate cancer (TTC) and overall survival (OS) of mHNPC patients by multivariate analysis. Moreover, we developed a novel prognostic model that consisted of significant prognostic factors.

**Results:**

Of the entire cohort, the median TTC and OS values were 18.3 and 67.5 months, respectively. HVD, GS 9‐10, and Hb ≤13.0 g/dL were independent poor prognostic factors for both TTC and OS. We developed a novel prognostic model which could stratify mHNPC patients into four risk groups according to the numbers of poor prognostic factors: group 1, LVD with low‐risk (LVD patients without GS 9‐10 and Hb ≤13.0 g/dL); group 2, LVD with high‐risk (LVD patients with GS 9‐10, Hb ≤13.0 g/dL, or both); group 3, HVD with low‐risk (HVD patients without GS 9‐10 with or without Hb ≤13.0 g/dL); and group 4, HVD with high‐risk (HVD patients with GS 9‐10 with or without Hb ≤13.0 g/dL). The median TTC and OS of groups 1, 2, 3, and 4 were 124.8, 36.4, 17.9, and 11.2 months, and 117.2, 94.2, 67.9, and 46.2 months, respectively. A significant difference in TCC and OS was found between all groups.

**Conclusion:**

We developed a prognostic model for mHNPC patients that consisted of CHAARTED risk classifications, GS, and Hb. Our prognostic model could significantly stratify the prognosis of patients with LVD and HVD into two groups each. This model might be a good reference for shared decision making between patients and physicians on the initial treatment for mHNPC.

## 1 INTRODUCTION

Prostate cancer is currently one of the most common cancers, as more than 1.3 million cases were newly diagnosed worldwide in 2018,^1^ and the incidence of prostate cancer has been particularly increasing in northeast Asian countries.^2^ One reason for the increase in the number of prostate cancer patients is the spread of prostate‐specific antigen (PSA) screening, which has led to earlier detection and a decrease in the mortality rate of prostate cancer.^3^, ^4^ However, there are still quite a few patients with distant metastases at first diagnosis, and appropriate treatment is required especially for these newly diagnosed (de novo) metastatic hormone‐naïve prostate cancer (mHNPC) patients. For mHNPC patients, androgen deprivation therapy (ADT), along with the upfront use of docetaxel or androgen receptor targeting agent (ARTA) including abiraterone acetate, apalutamide, and enzalutamide, is now the standard of care (SOC).^5^, ^6^, ^7^, ^8^ In addition, Phase III Systemic Therapy for Advanced or Metastatic Prostate cancer: Evaluation of Drug Efficacy (STAMPEDE) trial arm H showed that local radiotherapy to the prostate improved the survival of mHNPC patients with a low‐tumor burden.^9^ Thus, local radiotherapy is considered a SOC for mHNPC patients with low tumor burden.

Currently, many treatment strategies for mHNPC patients are available, but the appropriate treatment remains unclear because those patients are known to have a wide spectrum of progression risks.^10^, ^11^, ^12^ Therefore, predicting the survival of mHNPC patients is important when making treatment strategies for them. In this study, we aimed to investigate the prognosis of Japanese patients newly diagnosed with mHNPC treated with primary ADT and to develop a novel prognostic model for these patients.

## 2 MATERIALS AND METHODS

### 2.1 Study population

We retrospectively identified 593 newly diagnosed Japanese de novo mHNPC patients with bone metastases. These patients were initially treated with ADT and were registered in the Metropolitan Prostate Cancer Group (MPCG) database between January 2004 and December 2015. Fifteen patients were excluded due to a lack of data and 578 patients were ultimately evaluated.

All patients had a histologically confirmed diagnosis of prostate adenocarcinoma. Metastatic sites were evaluated by computed tomography (CT) and bone scan using 99‐technetium methylene diphosphonate/hydroxymethylene diphosphonate before any treatment. ADT was carried out by medical castration with luteinizing hormone‐releasing hormone (LHRH) agonist or antagonist combined with bicalutamide (80 mg daily, approved dose in Japan). Patients did not receive upfront abiraterone, apalutamide, enzalutamide, or docetaxel for mHNPC. Clinical data including patients’ age, PSA, Gleason scores (GS), hemoglobin (Hb), CT findings, bone scan findings, time to castration‐resistant prostate cancer (TTC), and overall survival (OS) for mHNPC were obtained from electronic medical records. GS was determined by a pathologist at each facility according to Gleason grading.^13^ Hb levels were examined prior to the start of ADT. The patients were classified into two survival risk groups defined by the ChemoHormonal Therapy Versus Androgen Ablation Randomized Trial for Extensive Disease in Prostate Cancer (CHAARTED) trial^14^: High‐volume disease (HVD) was defined as the presence of visceral metastases or ≥4 bone lesions with ≥1 beyond the vertebral bodies and pelvis and low‐volume disease (LVD) was defined as a non‐HVD.^6^ Metastatic castration‐resistant prostate cancer (mCRPC) was defined according to PCWG‐2.^15^ After progression to the mCRPC state, all patients were administered with an LHRH agonist or antagonist continuously and subsequently treated according to each attending physician’s treatment strategy. Flutamide, oral steroids, estramustine phosphate, and docetaxel were mainly used for those mCRPC patients until 2014. Since 2014, ARTA including abiraterone acetate and enzalutamide, docetaxel, and cabazitaxel was mainly used for those mCRPC patients; in addition, radium‐223 was used since 2016 as appropriate according to each attending physician’s choice. Bone‐modifying agents such as denosumab and zoledronic acid were also used for mCRPC patients according to the attending physicians’ choice. In the terminal stage, palliative therapy, pain control with morphine, and palliative external‐beam radiation were used as appropriate.

### 2.2 Prognostic factors and prognostic model

We evaluated three clinical factors, namely, CHAARTED risk classifications^6^ (HVD vs LVD), GS (9‐10 vs ≤8), and Hb (≤ 13.0 g/dL vs >13.0 g/dL) as prognostic significance for TTC and OS of mHNPC patients by univariate and multivariate analyses. Moreover, we developed a novel prognostic model that consisted of statistically significant prognostic factors.

### 2.3 Statistical analysis

A Kaplan‐Meier (KM) product‐limit estimator was used to assess the TTC and OS of mHNPC patients. A generalized Wilcoxon test was used to analyze the differences in TTC and OS between the groups. For detecting the prognostic factors for TCC and OS, univariate and multivariate analyses were performed using a Cox proportional hazards regression model.

We derived relative risks and 95% confidence intervals (95% CI). All tests were two‐sided; an alpha value of .05 was considered significant. The statistical software “EZR” (version 1.40; Saitama Medical Center, Jichi Medical University, Saitama, Japan), a graphical user interface for R (version 3.5.2; The R Foundation for Statistical Computing, Vienna, Austria),^16^ was used for drawing the KM curve. Other analyses were all conducted using IBM SPSS Statistics software for Windows version 26 (IBM Corp., Armonk, NY, USA).

## 3 RESULTS

### 3.1 Patients’ characteristics

Patients’ characteristics are shown in Table 1. The median age was 72 (range, 42‐92) years. The median initial PSA level was 267 (range, 1‐25 000) ng/mL, and 311 patients (53.8%) had GS 9‐10 and 267 patients (46.2%) had GS ≤8. The median Hb level was 13.0 (range, 6.5‐18.3) g/dL. There were 187 patients (32.4%) and 391 patients (67.6%) with LVD and HVD, respectively. Sixty patients (10.4%) had visceral metastases. Detailed information about the sites of visceral metastases was missing. The median observation time was 44.4 months.

**TABLE 1:** Patients’ characteristics (n = 578)

Median age, years (range)	72 (42‐92)
Median initial PSA levels, ng/mL (range)	267 (1‐25 000)
*Gleason scores, n (%)*	
≤7	90 (15.6)
8	177 (30.6)
9	239 (41.3)
10	72 (12.5)
Median hemoglobin, g/dL	13.0 (6.5‐18.3)
*CHAARTED risk classifications, n (%)*	
Low‐volume disease	187 (32.4)
High‐volume disease	391 (67.6)
*Visceral metastases, n (%)*	
No	518 (89.6)
Yes	60 (10.4)

Abbreviation: PSA, prostate‐specific antigen.

### 3.2 Clinical factors with prognostic significance for TTC and OS

**FIGURE 1:**
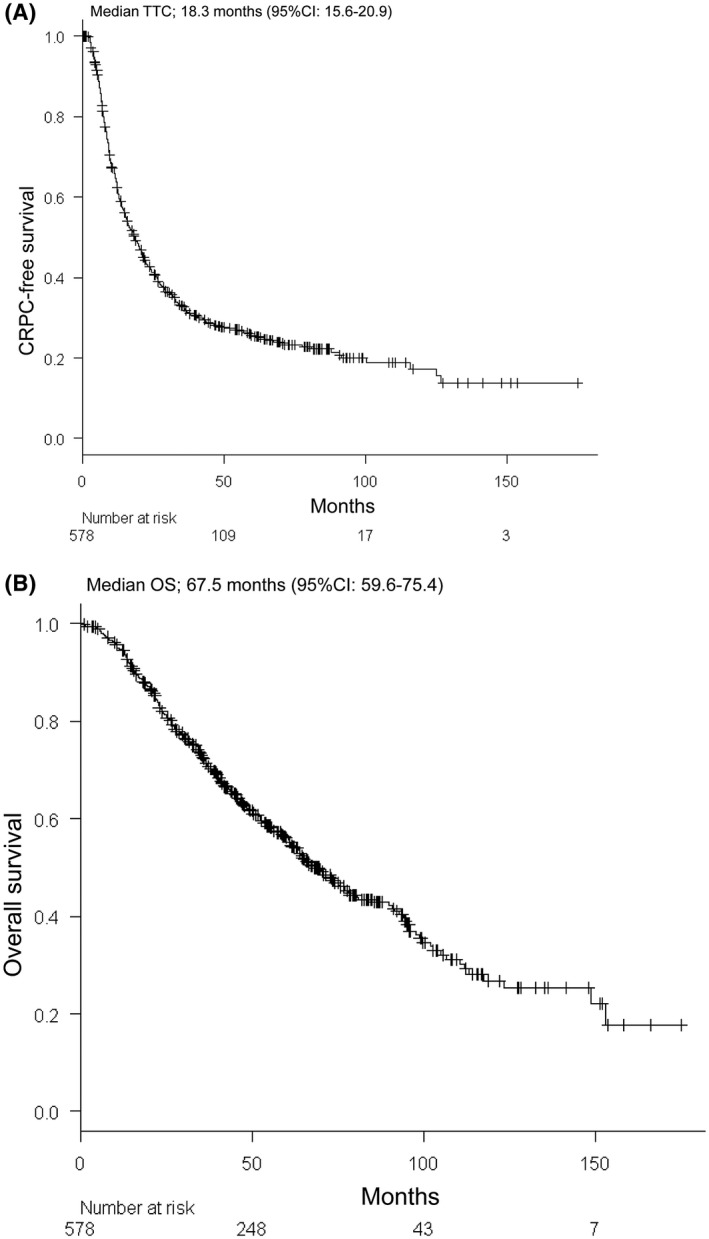
Kaplan‐Meier curve for time to castration‐resistant prostate cancer (TTC) and overall survival (OS) of the entire cohort of metastatic hormone‐naïve prostate cancer (mHNPC) patients. (A) Kaplan‐Meier curve for TTC. (B) Kaplan‐Meier curve for OS

**FIGURE 2:**
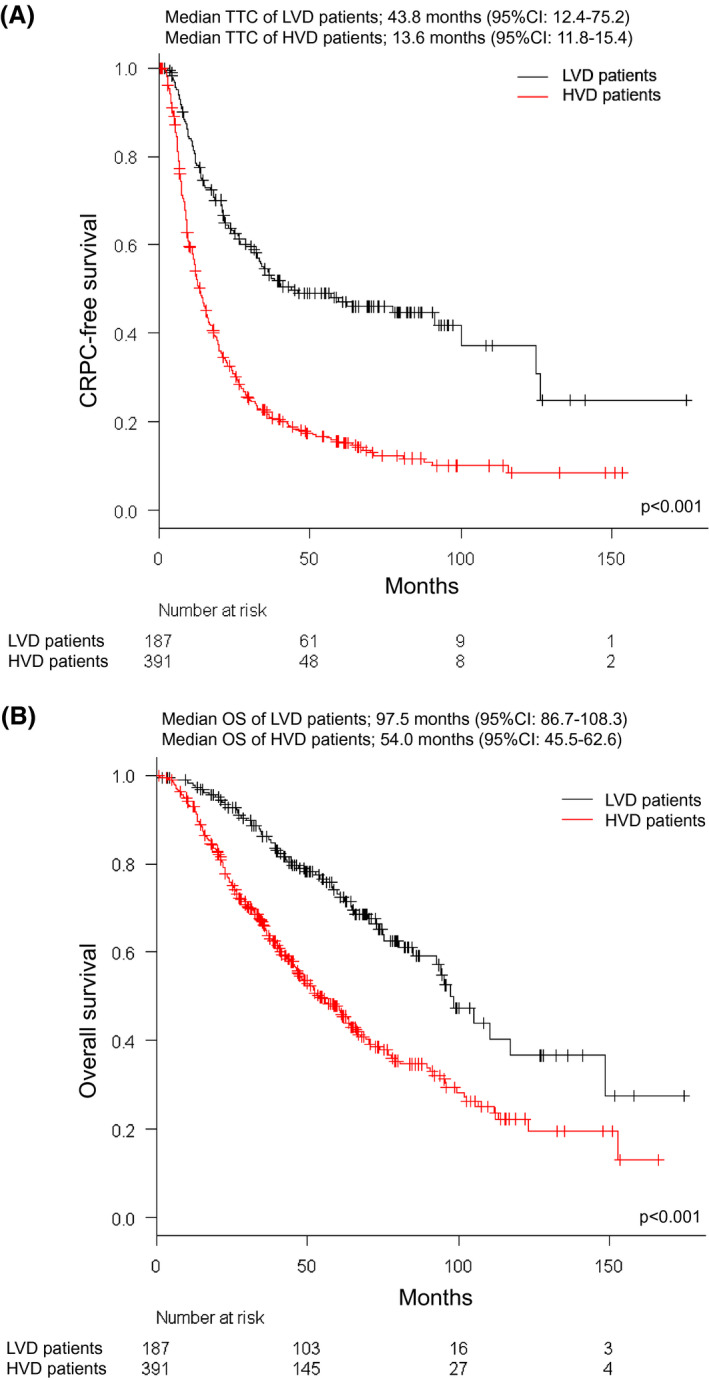
aplan‐Meier curves for time to castration‐resistant prostate cancer (TTC) and overall survival (OS) of metastatic hormone‐naïve prostate cancer (mHNPC) patients according to CHAARTED risk classifications. (A) Kaplan‐Meier curve for TTC. The black line and red line show the survival curve of TTC in mHNPC patients with low‐volume disease (LVD) and high‐volume disease (HVD), respectively. (B) Kaplan‐Meier curve for OS. The black line and red line show the survival curve of OS in mHNPC patients with LVD and HVD, respectively

The KM curves for TTC and OS of the entire cohort are shown in Figures 1A,B. The median TTC and OS were 18.3 months (95% CI 15.6‐20.9) and 67.5 months (95% CI 59.6‐75.4), respectively. Figures 2A,B show the KM curve for TTC and OS according to the CHAARTED risk classifications. The median TTC of LVD and HVD patients were 43.8 months (95% CI 12.4‐75.2) and 13.6 months (95% CI 11.8‐15.4), respectively (*P* < .001). The median OS of LVD and HVD patients was 97.5 months (95% CI 86.7‐108.3) and 54.0 months (95% CI 45.5‐62.6), respectively (*P* < .001).

**FIGURE 3:**
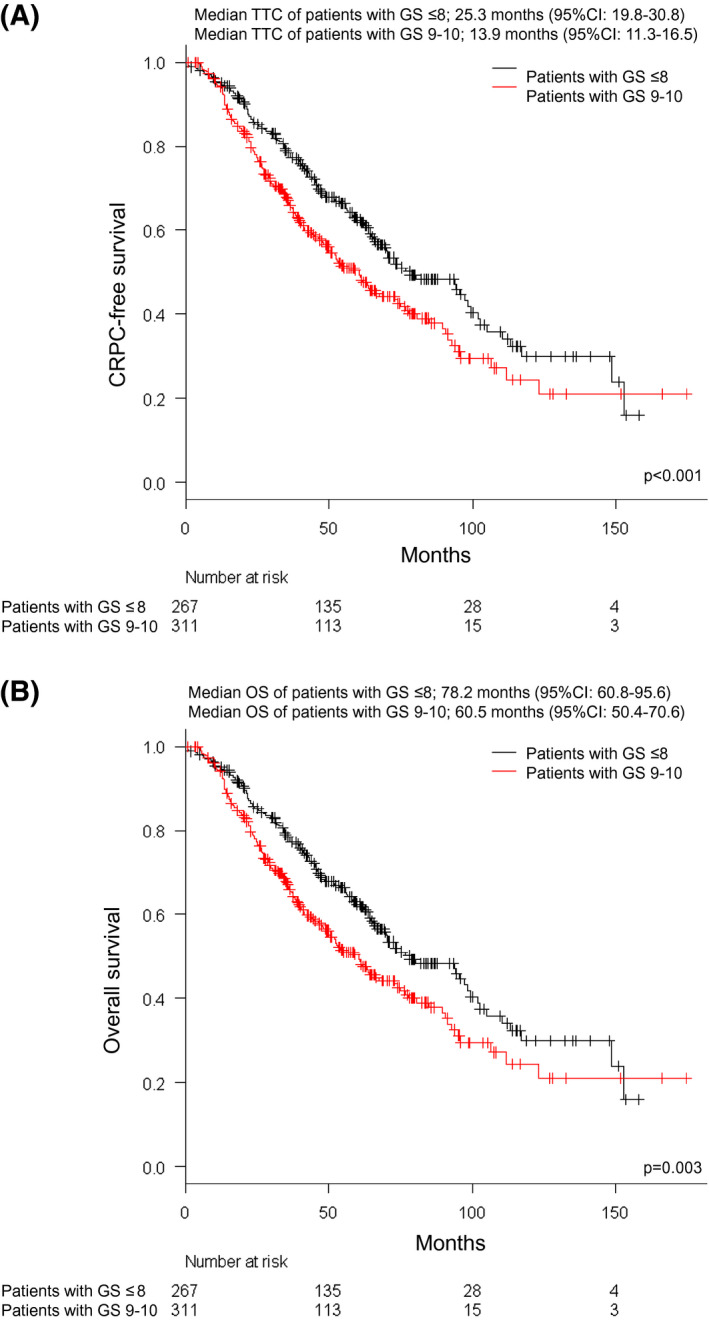
Kaplan‐Meier curves for time to castration‐resistant prostate cancer (TTC) and overall survival (OS) of metastatic hormone‐naïve prostate cancer (mHNPC) patients according to Gleason scores (GS). (A) Kaplan‐Meier curve for TTC. The black line and red line show the survival curve of TTC in mHNPC patients with GS ≤8 and GS 9‐10, respectively. (B) Kaplan‐Meier curve for OS. The black line and red line show the survival curve of OS in mHNPC patients with GS ≤8 and GS 9‐10, respectively

Figures 3A and 3B show the KM curve for TTC and OS according to GS. The median TTC of patients with low GS (≤8) and high GS (9‐10) was 25.3 months (95% CI 19.8‐30.8) and 13.9 months (95% CI 11.3‐16.5), respectively (*P* < .001).

The median OS of patients with high GS (≤8) and low GS (9‐10) was 78.2 months (95% CI 60.8‐95.6) and 60.5 months (95% CI 50.4‐70.6), respectively (*P* < .001).

**FIGURE 4:**
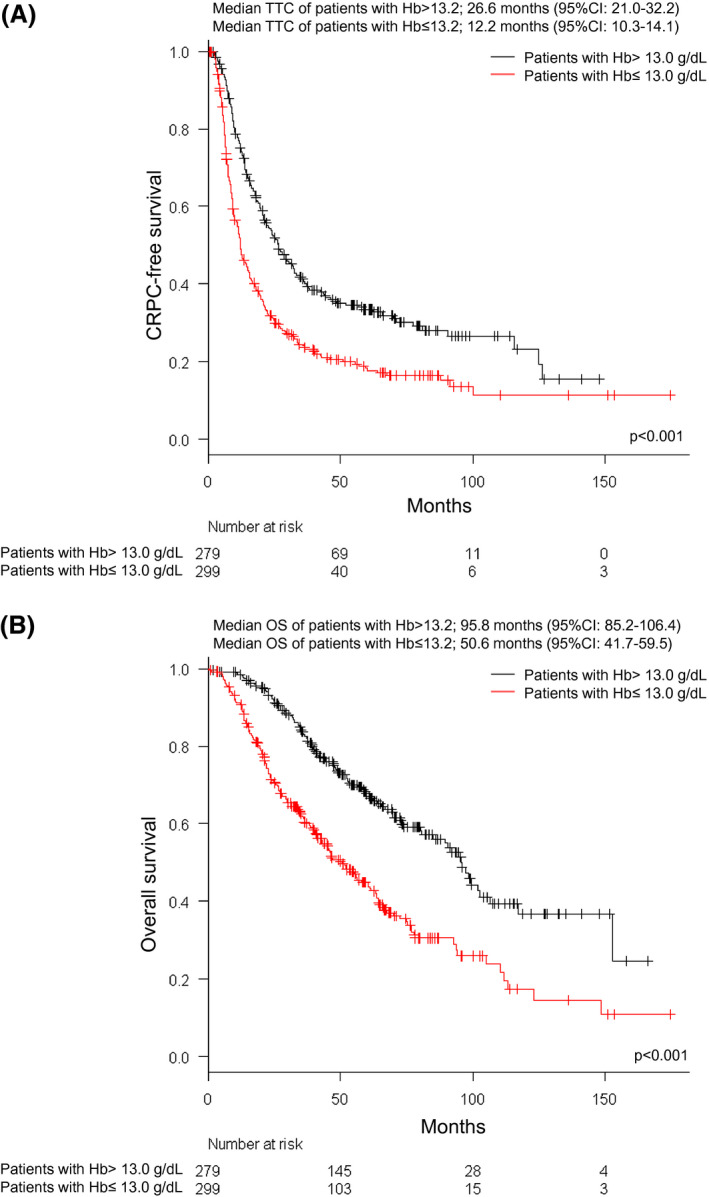
Kaplan‐Meier curves for time to castration‐resistant prostate cancer (TTC) and overall survival (OS) of metastatic hormone‐naïve prostate cancer (mHNPC) patients according to hemoglobin (Hb) levels. (A) Kaplan‐Meier curve for TTC. The black line and red line show the survival curve of TTC in mHNPC patients with Hb >13 and Hb ≤ 13 g/dL, respectively. (B) Kaplan‐Meier curve for OS. The black line and red line show the survival curve of OS in mHNPC patients with Hb >13 and Hb ≤ 13 g/dL, respectively

Figures 4A and 4B show the KM curve for TTC and OS according to Hb.

The median TTC of patients with high Hb (>13.0 g/dL) and low Hb (≤13.0 g/dL) was 26.6 months (95% CI 21.0‐32.2) and 12.2 months (95% CI 10.3‐14.1), respectively (*P* < .001). The median OS of patients with high Hb and low Hb was 95.8 months (95% CI 85.2‐106.4) and 50.6 months (95% CI 41.7‐59.5), respectively (*P* < .001).

Results of the univariate and multivariate analyses of clinical factors for predicting TTC and OS of mHNPC patients are shown in Tables 2 and 3. The univariate and multivariate analyses for predicting TTC showed that CHAARTED risk classifications, GS, and Hb were all significant prognostic factors. Similarly, the univariate and multivariate analyses for predicting OS showed that CHAARTED risk classifications, GS, and Hb were all significant prognostic factors. In the results, those three clinical factors, namely, HVD, GS9‐10, and Hb≤13.0 g/dL, were found to be independent significant poor prognostic factors for both TTC and OS.

**TABLE 2:** Univariate and multivariate analyses for predicting time to castration‐resistant prostate cancer

Variables		Univariate analysis			Multivariate analysis		
		HR	95% CI	*P* value	HR	95% CI	*P* value
CHAARTED risk classifications	HVD vs LVD	2.53	2.01‐3.19	<.001	2.35	1.86‐2.97	<.001
Gleason score	9‐10 vs ≤8	1.53	1.25‐1.85	<.001	1.71	1.41‐2.08	<.001
Hemoglobin	≤13 g/dL vs >13	1.73	1.42‐2.10	<.001	1.51	1.25‐1.84	<.001

Abbreviations: CI, confidence interval; HR, hazard ratio; HVD, high‐volume disease; LVD, low‐volume disease.

**TABLE 3:** Univariate and multivariate analyses for predicting the overall survival

Variables		Univariate analysis			Multivariate analysis		
		HR	95% CI	*P* value	HR	95% CI	*P* value
CHAARTED risk classifications	HVD vs LVD	2.18	1.64‐2.88	<.001	2.00	1.51‐2.65	<.001
Gleason score	9‐10 vs ≤8	1.43	1.13‐1.81	.003	1.38	1.10‐1.76	.007
Hemoglobin	≤13 g/dL vs >13	2.18	1.70‐2.78	<.001	2.06	1.61‐2.63	<.001

Abbreviations: CI, confidence interval; HR, hazard ratio; HVD, high‐volume disease; LVD, low‐volume disease.

### 3.3 Development of the prognostic model for TTC and OS

From those prognostic factors, we developed a prognostic model for TTC and OS of mHNPC patients. We stratified the entire cohort into four groups according to the number of poor prognostic factors:

Group 1, LVD with low‐risk (LVD patients without GS 9‐10 and Hb ≤13.0 g/dL);
Group 2, LVD with high‐risk (LVD patients with GS 9‐10, Hb ≤13.0 g/dL, or both);
Groups 3, HVD with low‐risk (HVD patients without GS 9‐10 with or without Hb ≤13.0 g/dL);
Group 4, HVD with high‐risk (HVD patients with GS 9‐10 with or without Hb ≤13.0 g/dL).

There were 57 (9.9%) patients in group 1, 130 (22.5%) patients in group 2, 167 (28.9%) patients in group 3, and 224 (38.7%) patients in group 4.

**FIGURE 5:**
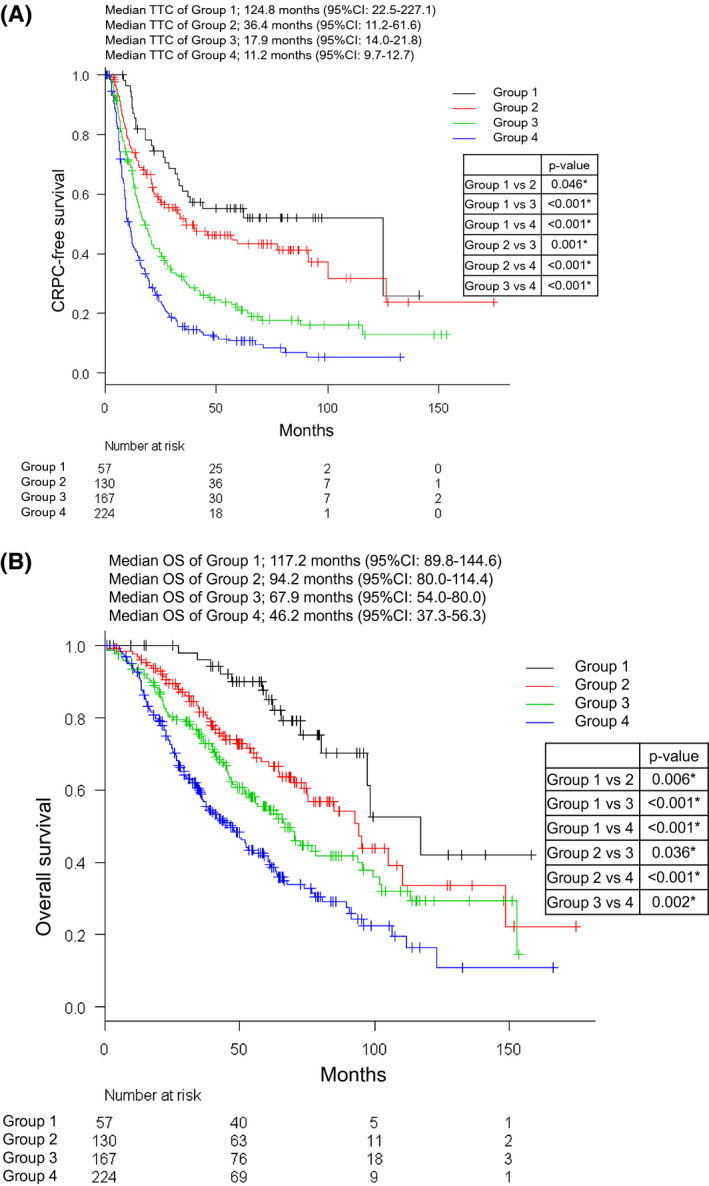
Kaplan‐Meier curves for time to castration‐resistant prostate cancer (TTC) and overall survival (OS) of metastatic hormone‐naïve prostate cancer (mHNPC) patients according to our prognostic model. (A) Kaplan‐Meier curve for TTC. The black, red, green, and blue lines show the survival curve of TTC in mHNPC patients in groups 1, 2, 3, and 4, respectively. (B) Kaplan‐Meier curve for OS. The black, red, green, and blue lines show the survival curve of OS in mHNPC patients in groups 1, 2, 3, and 4, respectively. Groups 1, 2, 3, and 4 indicate low‐volume disease (LVD) with low‐risk, LVD with high‐risk, high‐volume disease (HVD) with low‐risk, and HVD with high‐risk, respectively

Figure 5A shows the KM curve for TTC according to our prognostic model. The median TTC in groups 1, 2, 3, and 4 were 124.8 (95% CI 22.5‐227.1), 36.4 (95% CI 11.2‐61.6), 17.9 (95% CI 14.0‐21.8), and 11.2 (95% CI 9.7‐12.7) months, respectively. A significant difference in TTC was found between all groups.

Figure 5B shows the KM curve for OS according to our prognostic model. The median OS in group 1, 2, 3, and 4 were 117.2 (95% CI 89.8‐144.6), 94.2 (95% CI 80.0‐114.4), 67.9 (95% CI 54.0‐80.0), and 46.2 (95% CI 37.3‐56.3) months, respectively. A significant difference in OS was found between all groups. The number of patients of cancer death out of all‐cause death was 11/14 (78.6%) in group 1, 36/49 (73.5%) in group 2, 68/83 (81.9%) in group 3, and 123/33 (92.5%) in group 4.

## 4 DISCUSSION

In this study, we developed a prognostic model for mHNPC patients that consisted of CHAARTED risk classifications, GS, and Hb. LVD and HVD were first suggested in the clinical Phase III CHAARTED trial.^6^ In the CHAARTED trial, the median OS of newly diagnosed mHNPC patients treated by ADT (control arm) with LVD and HVD was 59.8 and 33.1 months, respectively.^17^ LATITUDE^18^ is a clinical phase III trial for LATITUDE high‐risk mHNPC patients; however, it included nearly 20% of LVD (number of patients treated with ADT alone as control arm: 110 [18.4%] of total 597). During this study, the median OS was not reached in LVD patients whereas it was 33.3 months in HVD patients. In our study, the median OS of LVD and HVD patients was 97.5 and 54.0 months, respectively. In our study, the median OS of LVD and HVD was 97.5 and 54.0 months, respectively. Our cohort had longer survival than those in the CHAARTED or LATITUDE trial. Recently, Akamatsu et al. also reported the survival of Japanese mHNPC patients treated with ADT.^11^ Their cohort included 100 patients (36.8%) with LVD and 172 patients (63.2%) with HVD; the median OS values were 131 and 53 months in LVD and HVD patients, respectively. Japanese patients with mHNPC could have longer survival than western patients. The reason for the longer survival among Japanese patients remain unclear; however, the difference in the sensitivity for hormone therapy between race^19^ or difference in socioeconomic status between nations^20^ could be suggested.

Various prognostic models for mHNPC patients have been reported. In 1988, Soloway et al. reported the extent of disease on bone scan (EOD) for predicting the survival of mHNPC patients.^21^ The 2‐year survival rates for EOD I to IV were 94%, 74%, 68%, and 40%, respectively, indicating that the EOD correlated with survival.

In 2003, Glass et al. also reported a prognostic model for predicting the survival of mHNPC patients.^22^ They analyzed data from 1076 patients included in the Phase III Southwest Oncology Group Study 8894 and developed a prognostic model for 5‐year survival that consisted of the site of bone metastases, performance status, PSA levels, and GS. This Glass scale^22^ could stratify the survival risk of mHNPC patients into three groups with statistical significance.

Recently, Akamatsu et al. developed a novel prognostic model for OS of Japanese mHNPC patients, which consisted of EOD, liver metastases, lactate dehydrogenase (LDH), and Gleason pattern 5.^11^ This model could stratify mHNPC patients into three risk groups.

Recently, Narita et al. reported a prognostic model for Japanese mHNPC patients.^10^ Their model consisted of GS, lymph node metastases, EOD, and GS and could stratify mHNPC patients into three risk groups. We previously reported a prognostic nomogram composed of five prognostic factors for Japanese mHNPC patients, namely, age, PSA levels, clinical T stage, EOD, and GS. This validated nomogram could estimate 1‐, 3‐, and 5‐year survival probability.^12^

Currently, ADT, ADT combined with docetaxel or ARTA (abiraterone acetate, apalutamide, and enzalutamide), and ADT plus local radiotherapy to the prostate are the SOC for mHNPC patients. In addition, an aggressive approach beyond SOC for LVD or oligometastatic prostate cancer (OMPC) patients was reported recently.^23^, ^24^ Tsumura et al. performed a retrospective analysis and reported that brachytherapy plus metastasis‐directed therapy (MDT) to newly diagnosed OMPC patients could improve CRPC‐free survival compared with brachytherapy alone.^23^ Through a retrospective case‐control study, Heidenreich et al. reported that cytoreductive prostatectomy for newly diagnosed OMPC patients could improve TTC.^24^

Cytoreductive prostatectomy for newly diagnosed mHNPC patients is being tested in nine prospective randomized clinical trials.^25^

As described above, many treatment strategies are available for newly diagnosed mHNPC patients currently; however, choosing the appropriate treatment for these patients according to various risks is important because they are known to have a wide spectrum of clinical progression risks.^12^

In our study, we developed a novel prognostic model that consisted of HVD, GS 9‐10, and Hb≤13.0, which demonstrated as statistically significant poor prognostic factors. All variables have been also reported as prognostic factors for mHNPC patients.^10^, ^11^, ^12^, ^26^ Although anemia’s etiology can be a disease other than prostate cancer, Beer et al. reported that anemia was associated with shorter survival among newly diagnosed mHNPC patients. This study hypothesized that anemia contributed to tumor hypoxia and induced resistance to ADT, which could explain why a low Hb level was identified as a poor prognostic factor.^26^

Our novel prognostic model might be the first model that could stratify finer survival risk of mHNPC patients based on LVD and HVD with two groups each, ie, LVD with low‐risk as group 1, LVD with high‐risk as group 2, HVD with low‐risk as group 3, and HVD with high‐risk as group 4. The median TTC of patients in groups 1, 2, 3, and 4 were 124.8, 36.4, 17.9, and 11.2 months, respectively, and a significant difference in TTC was found between all groups. Similarly, the median OS of patients in groups 1, 2, 3, and 4 were 117.2, 94.2, 67.9, and 46.2 months, respectively, and a significant difference in OS was found between all groups.

Subsequently, when selecting the treatment strategies from many options for newly diagnosed mHNPC patients, CHAARTED risk classifications alone could not be enough for choosing the appropriate treatment. Our novel prognostic model could be a good reference for shared decision making between patients and physicians when considering initial treatment strategies for mHNPC. According to our results, mHNPC patients with HVD and high‐risk (group 4) would benefit from intensive therapy.

This study had some limitations. First, this was a retrospective study with relatively few patients and a short observation time. As mentioned above, serum LDH levels have reported as significant prognostic factors. However, we could not analyze the correlation between LDH levels and the prognosis of mHNPC patients because the LDH assay kit was different for each facility. Besides, pain at baseline has also been reported as a significant prognostic factor among patients with mHNPC.^27^ Unfortunately, this information was not included in our MPCG database.

Second, the treatment for CRPC after primary ADT differs by period, as new chemotherapies, ARTA, and bone modifying agents have been used in common daily practice. Unfortunately, there were no data about mCRPC therapies in our database. In Japan, docetaxel was approved before 2014 (approved in 2008). While ARTA, cabazitaxel, and radium‐223 were approved after 2014. It was assumed that after developing mCRPC, the earlier cohort (diagnosed from 2004 to 2009) might have only received docetaxel as a life‐prolonging agent, while the later cohort (diagnosed from 2010 to 2015) might have received ARTA, cabazitaxel, and radium‐223 in addition to docetaxel. For evaluating the impact of mCRPC therapies, we evaluated KM curves for earlier and later cohorts separately. There were 212 and 366 patients in earlier and later cohorts, respectively. Twenty‐six patients (12.3 %) in the early cohort and 213 (58.2%) patients in the later cohort developed mCRPC after 2014. Figures S1A and B display the KM curves for OS in earlier and later cohorts, respectively. The lack of data for mCRPC therapies could influence our prognostic model for predicting OS; however, these results demonstrated that our prognostic model could also classify OS in both cohort patients and suggested that the impact of mCRPC therapies may not have a significant influence on our model.

Third, our results were based on the data from Japanese patients; thus, our results may not be applicable to western patients. Finally, next‐generation imaging (NGI) for prostate cancer detection such as prostate‐specific membrane antigen‐positron emission tomography (PET), choline PET, and fluciclovine PET, has been developed recently, and these techniques are more sensitive to metastases detection than conventional imaging such as CT and bone scan.^28^ Moreover, as applications of NGI continue to increase, the treatment of strategy for patients with few metastases has changed. MDT based on NGI is expected to improve survival. Our prognostic model was developed based on clinical information obtained by conventional imaging including CT, bone scan, and magnetic resonance imaging. When NGI would be clinically applied in the near future, a new prognostic model for NGI should be developed.

In conclusion, our novel prognostic model might be the first model that could stratify the finer survival risk of mHNPC patients based on LVD and HVD, with two groups each. This prognostic model might be a good reference for shared decision making between patients and physicians of the initial treatment for mHNPC. To confirm our results, a prospective study is warranted.

## CONFLICT OF INTEREST

The authors declare that they have no competing interests

## CONSENT FOR PUBLICATION

We obtained IRB approval, including publication allowance.

## ETHICS APPROVAL AND CONSENT TO PARTICIPATE

This study was approved by the IRB of Yokohama City University Medical Center (B180500019).

## Supporting information

Fig S1‐S2

## Data Availability

Due to ethical restrictions, the raw data underlying this paper are available upon request to the corresponding author.
