# Supplementary material for: A novel prognostic model for Japanese patients with newly diagnosed bone‐metastatic hormone‐naïve prostate cancer
Source: BJUI Compass. 2020 Sep 18;2(2):105–14. doi: 10.1002/bco2.46 (PMC8988841; doi:10.1002/bco2.46)
Supplement: Supplementary file 1 — Fig S1‐S2 [file BCO2-2-105-s001.pptx]

## Slide 1
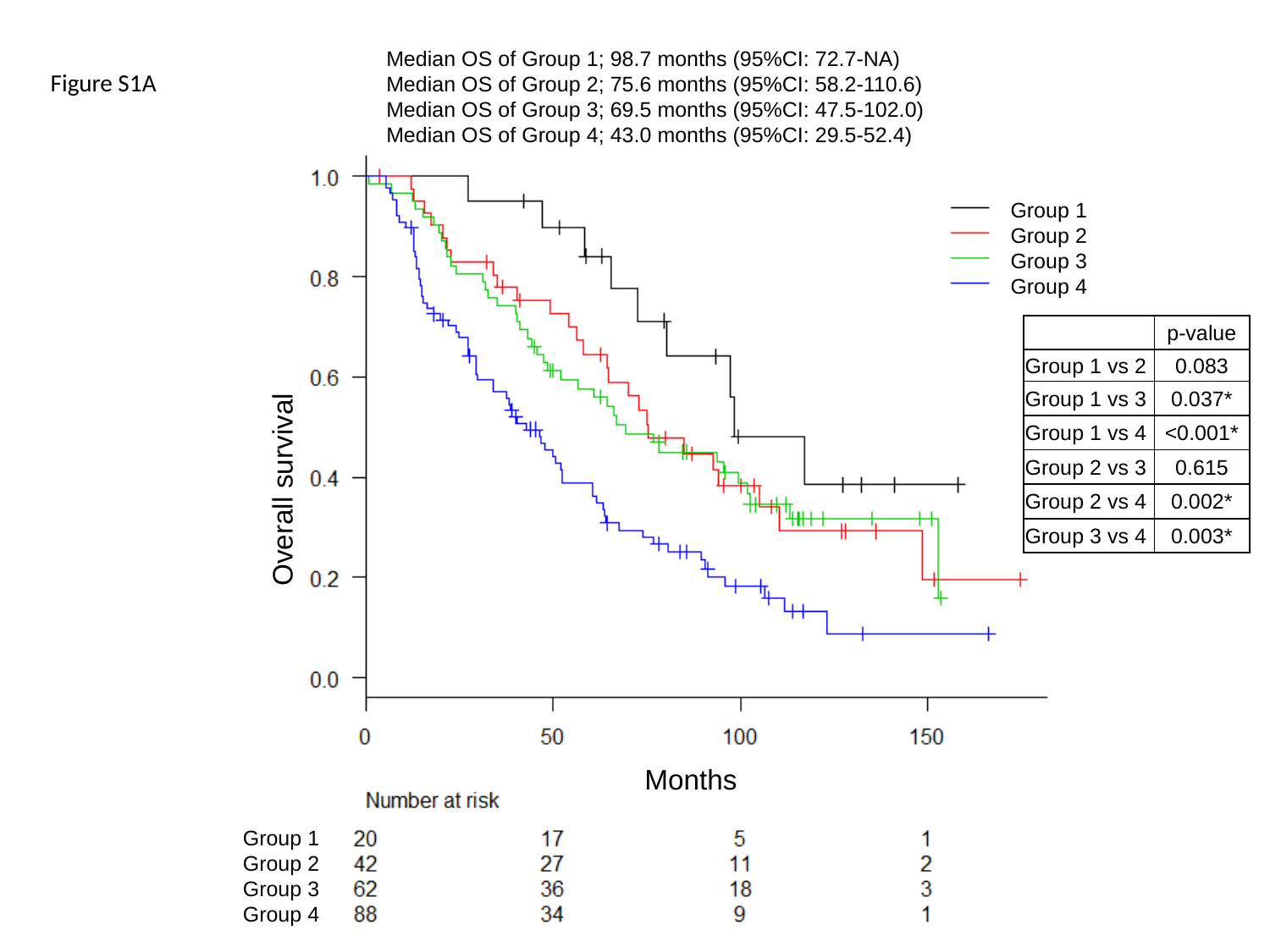

Median OS of Group 1; 98.7 months (95%CI: 72.7-NA)
Median OS of Group 2; 75.6 months (95%CI: 58.2-110.6)
Median OS of Group 3; 69.5 months (95%CI: 47.5-102.0)
Median OS of Group 4; 43.0 months (95%CI: 29.5-52.4)
Figure S1A
Group 1
Group 2
Group 3
Group 4
| | p-value |
| --- | --- |
| Group 1 vs 2 | 0.083 |
| Group 1 vs 3 | 0.037\* |
| Group 1 vs 4 | <0.001\* |
| Group 2 vs 3 | 0.615 |
| Group 2 vs 4 | 0.002\* |
| Group 3 vs 4 | 0.003\* |
Overall survival
Months
Group 1
Group 2
Group 3
Group 4

## Slide 2
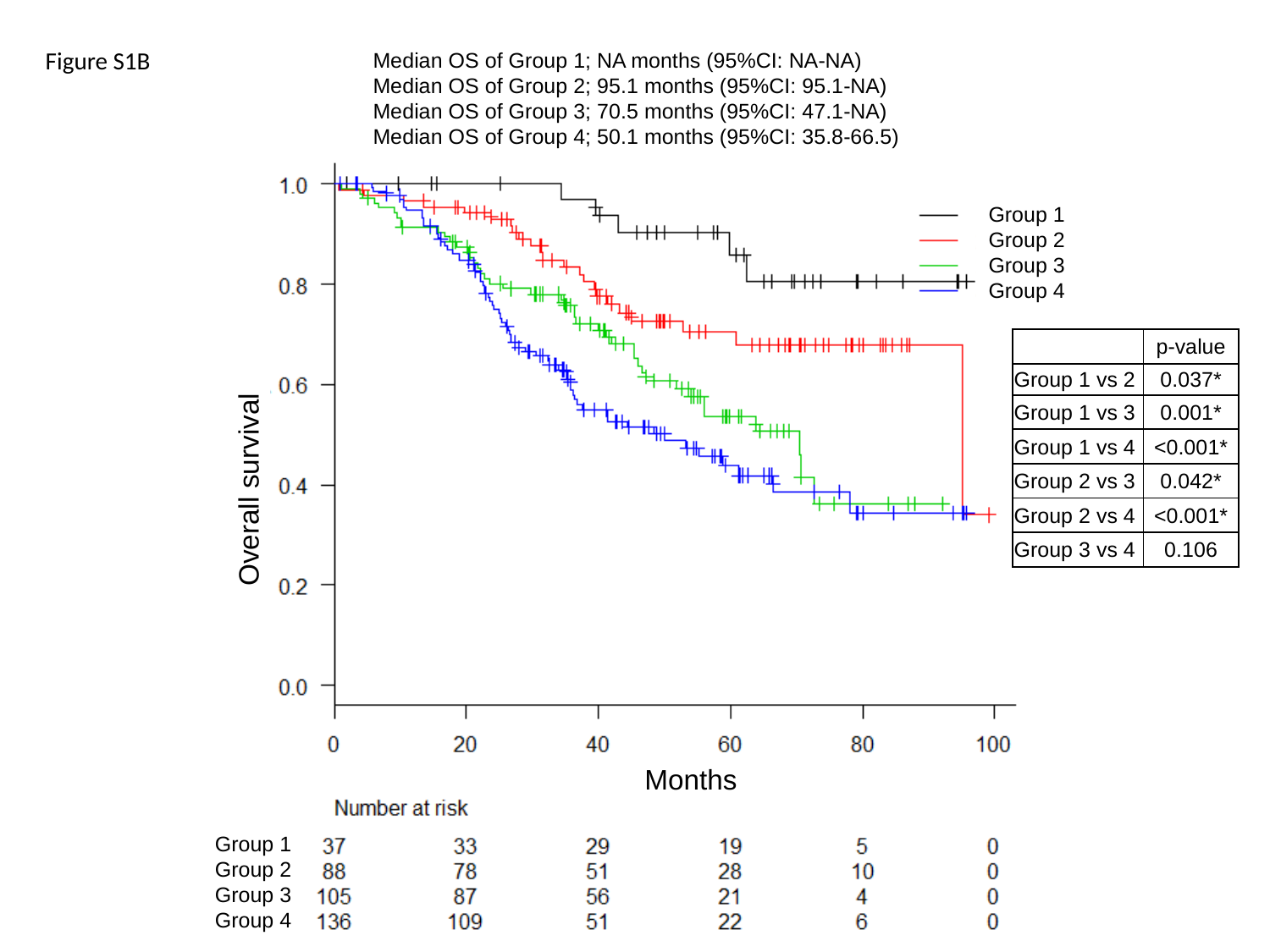

Figure S1B
Median OS of Group 1; NA months (95%CI: NA-NA)
Median OS of Group 2; 95.1 months (95%CI: 95.1-NA)
Median OS of Group 3; 70.5 months (95%CI: 47.1-NA)
Median OS of Group 4; 50.1 months (95%CI: 35.8-66.5)
Group 1
Group 2
Group 3
Group 4
| | p-value |
| --- | --- |
| Group 1 vs 2 | 0.037\* |
| Group 1 vs 3 | 0.001\* |
| Group 1 vs 4 | <0.001\* |
| Group 2 vs 3 | 0.042\* |
| Group 2 vs 4 | <0.001\* |
| Group 3 vs 4 | 0.106 |
Overall survival
Months
Group 1
Group 2
Group 3
Group 4
